# Resolving network clusters disparity based on dissimilarity measurements with nonmetric analysis of variance

**DOI:** 10.1016/j.isci.2023.108354

**Published:** 2023-10-28

**Authors:** Alina Malyutina, Jing Tang, Ali Amiryousefi

**Affiliations:** 1 Research Program in Systems Oncology, Faculty of Medicine, University of Helsinki, 00014 Helsinki, Finland; 2 Laboratory of Systems Pharmacology, Harvard Medical School, 200 Longwood Avenue, Boston, MA 02115, USA

**Keywords:** Biological sciences, Biocomputational method, Statistical computing

## Abstract

Classic ANOVA (cA) tests the explanatory power of a partitioning on a set of objects. More fit for clusters proximity analysis, nonparametric ANOVA (npA) extends to a case where instead of the object values themselves, their mutual distances are available. However, extending the cA applicability, the metric conditions in npA are limiting. Based on the central limit theorem (CLT), here we introduce nonmetric ANOVA (nmA) that by relaxing the metric properties between objects, allows an ANOVA-like statistical testing of a network clusters disparity. We present a parametric test statistic which under the null hypothesis of no differences between the competing clusters means, follows an exact *F*-distribution. We apply our method on three diverse biological examples, discuss its parallel performance, and note the specific use of each method tailored by the inherent data properties. The R code is provided at github.com/AmiryousefiLab/nmANOVA.

## Highlights

• Nonmetric ANOVA renders a parametric test based on objects’ dissimilarities
• Disparity significance of network clusters with dissimilarities is quantified
• Maximizing observed statistic yields most orthogonal nonmetric network partitioning

## Graphical abstract

**Figure:**
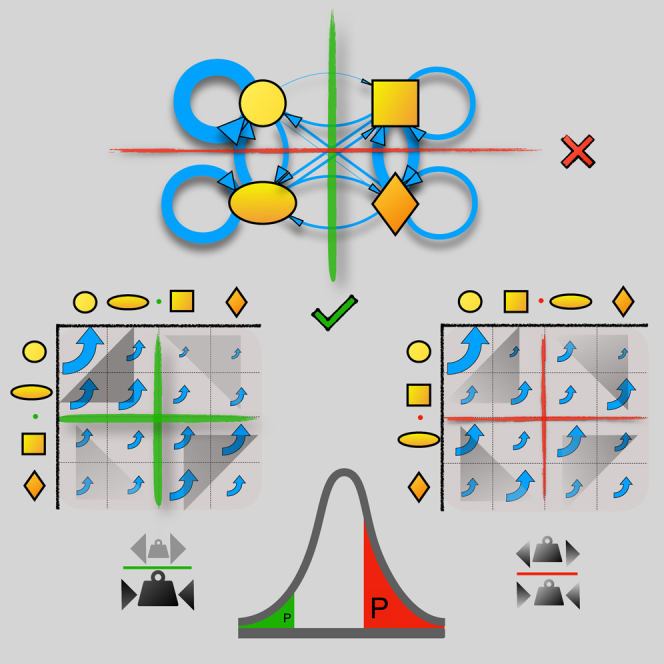

## Introduction

Classic ANOVA (cA),^1^ stands as a renown generic statistical tool for testing mean similarity of different groups.^2^^,^^3^ Since its introduction, cA has been developed to take into account various aspects of the experimental design and properties of observed variables from unbalanced sampling^4^ to non-Gaussian residuals by Kruskal-Wallis type of nonparametric testing.^5^^,^^6^^,^^7^ A special case where the reference point to which the measurement of objects is not available but still one is able to collect all the pairwise distances between them, caught considerable attention by a series of work.^8^^,^^9^ In this scenario, the emphasis has been put on the use of distances between responses and derivation of a permutative method for forming a pseudo-*F* hypothesis testing statistic (hence nonparametric ANOVA; npA) based on the distances of the responses.^9^^,^^10^ In npA, the original sum of squared distances of responses from their means was replaced by the mean sum of square distances between responses. This was shown to lead into a variance decomposition that equates the case where the observation vectors themselves are considered (Data S3.2). While most experimental variables could be measured with metric functions, there are cases where only nonmetric dissimilarities are obtainable. However, lacking the appropriate method tailored for this type of input is leading to different types of data coercion to enable the downstream analysis. Specifically, while the transformation of nonmetric outputs to Euclidean^11^ renders the npA operability, it is leading to invalid judgments due to compressing and deforming the data beyond sufficiency. The failure to appropriately acknowledge the data inherency is driving the misjudgment led by the deficient data coercion. Network graphs rising in multiple biological studies for example,^12^^,^^13^ could exhibit the nonsymmetrical measurements between the nodes, lack the edge between two, or even contain a self-looped nodes, all of which being cases where the metric properties are negated (Data S1). Each of these nonmetric scenarios should then be represented with a dissimilarity matrix between the nodes (rather than being metric-forced) and so demand a statistical framework that could incorporate this input as it is.

Here, based on the central limit theorem (CLT) and harnessing the natural relationship between statistical distributions,^14^ we generalize the cA method to a nonmetric case by introducing a nonmetric ANOVA (nmA). This development allows the direct integration of the nonmetric data input and unlike npA, provides a parametric p value based on the theoretical distribution of an *F*-statistic that under null hypothesis of means similarity between partitioning classes, follows the exact *F*-distribution. This enables the hypothesis testing for the experimental settings with nonmetric outputs. As we demonstrate with three diverse biological case studies, the nmA provides a statistical testing solution that allows the integration of the unmodulated dissimilarities input into the test statistic, downstream testing mechanics, and a resultant p value.

The following section stretches out the theoretical foundation of nmA and covers its major properties in different hypothetical settings. This will be followed by application of the nmA in three real case studies where the underlying data are not following any known metric function. Finally, we discuss the merit and limitations of nmA and conclude the usability sphere of this method.

## Results

The following subsection provides a background account for the nonmetric scenarios and their relevance. This will be continued with formal representation of the nmA and its hypothesis testing framework. Finally, followed by three biological examples, the last subsection presents the general properties of the nmA such as sensitivity, asymptotic behavior, and presents its appropriate placement in relation to the conventional methods.

### Beyond metric

In many practical cases, the level of dissimilarities expressed between items does not follow the metric properties.^15^ Based on triangle equality, the simultaneous affirmation of all derived equations commonly referred to as identity, symmetry, and subadditivity (Data S3) is necessary for a set of dissimilarities to be defined as metric.^16^ The negation of each to all of these equations (and hence invalidating the metric definitions) is extensively classified.^17^ For example, the term *semimetric* denotes scenarios where the triangle inequality fails to hold in general, while a *pseudometric* refers to cases where the forward condition of the identity property fails^18^; the case where two distinct objects have a zero distance. Referring to the network analogy, this equates the cases with two distinct nodes with a complete connectivity such that the information in either of nodes is directly available in the other. Metametric, on the other hand, is an opposite case where the distance between two identical items can be more than zero; a self-looped node that might lose the information received,^19^ and lastly, *quasimetric* refers to cases where the symmetry condition is distorted such that the distance from objects *A* to *B* is not necessarily the same as distance from *B* to *A*^20^; such as inequality of the edges and hence unbalanced information flow between two nodes in a directed network. These violations of the metric properties in general provide a ground for assessing cases where the measurements do not exhibit metric properties.^21^ Next to the classic invention of nonmetric methods like Kruskal multidimensional rank test^22^ and Mantel permutation tests,^23^ the prevalence of the nonmetric data has led to considerable advancement in other statistical domains as a proportionate response for the existing demand. For example, decision trees finding based on nonmetric data provides a robust tree optimization, and partial least square and generalized linear models offer solutions for the regression fitting for nonmetric outputs.^24^^,^^25^^,^^26^^,^^27^ In alignment to these advancement and reference to the partitioning scenarios, here we extend the cA model to nmA. This extension allows for example, obtaining the p value for each of the bifurcating nodes on the decision tree and assessing the multipartitism of a generic network. Also as a meaningful add to the nonmetric generalized linear models, nmA permits a similar hypothesis testing between objects in different partitions. As such, given the dissimilarity measurements, the resultant p value of this method could also be used as a yardstick for finding the most distinct partitioning underlying a set of objects.^28^

### Hypothesis testing with nmA

Consider an experiment where the independent variable imposes a partitioning on the responses and the task is to assess the significance of this partitioning. Similar to the nonparametric model (Data S3.2), the partitions are assumed to be exchangeable, but the dissimilarities between responses are now assumed to be any type of dissimilarities which are not necessarily outputs of a defined metric function.

For any α and β in range of *N* number of objects, define $\delta_{\alpha \beta }$ as the outcome of the dissimilarity function $\delta \left(y_{\alpha },y_{\beta }\right)$ where y is either a scalar or a vector of the interested response. Collecting all the pairwise dissimilarities in a square matrix $\Delta_{N\times N}=\left\{\delta_{\alpha \beta }\right\}$ forms the *dissimilarity* matrix between objects as the counterpart of the *distance* matrix in npA (Data S3.2). Furthermore, we assume that the dataset partitioned into *g* groups, each containing $n_{j|j=1,\ldots ,g}$ items such that $N=\underset{}{\sum_{j}}n_{j}$ constitute the total number of objects. For each $j,j^{\prime }=1,2,\ldots \text{,}g$ and $j\neq j^{\prime }$, let us denote $\bar{\delta_{jj}}$ and $\bar{\delta_{jj^{\prime }}}$ as the mean dissimilarity measures of the diagonal and off-diagonal sub-matrices of the Δ that are indexed distinctively for *j*th group. We denote $N_{off}$ as the number of elements in all off-diagonal sub-matrices (*between*), and $N_{diag}$ as the number of elements in all diagonal sub-matrices (*within*). Let us define ${\bar{\delta_{jj^{\prime }}}}^{prop}$ as the mean of a random $N_{diag}/N_{off}$ portion of dissimilarity measures in all *within* sub-matrices. We further restrict ${\bar{\delta_{jj^{\prime }}}}^{prop}$ so that for any *j* and $j^{\prime }$, it does not include any element from neither of these partitions. The latter is needed to ensure the statistical independence of the numerator and denominator of the (Equation 1). Under the null hypothesis of similarity of all partition means, $H_{0}:\forall j,j^{\prime }\in \left(1,\ldots ,g\right),\mu_{jj^{\prime }}=\mu$, and hence ${\bar{\delta }}_{11}={\bar{\delta }}_{12}={\bar{\delta }}_{12}={\bar{\delta }}_{22},\ldots \text{,}{\bar{\delta }}_{gg}={\bar{\delta }}_{W}$, the $F_{nm}$ as presented in the following equation is following the *F*-distribution with $g^{2}-g$ and g−1 degrees of freedom;

(Equation 1) $$\begin{array}{cc} F_{nm}=\frac{SNM_{B_{\delta }}/g}{SNM_{W_{\delta }}} & {∼}_{H_{0}}\;F{\left(x\right)}_{g^{2}-g,g-1}\text{,} \end{array}$$

where $SNM_{B_{\delta }}$ and $SNM_{W_{\delta }}$ are the *sums of squared normalized means* of dissimilarity values *between* and *within* partitioning groups defined as

(Equation 2) $$\begin{array}{c} SNM_{W_{\delta }}={\sum_{j=1}^{g}\left(n_{j}\left({\bar{\delta }}_{jj}-{\bar{\delta }}_{W}\right)\right)}^{2}\\ SNM_{B_{\delta }}=\sum_{j=1}^{g}\sum_{j^{\prime }\neq j}^{g}{\left(\frac{\left({\bar{\delta }}_{jj^{\prime }}-{{\bar{\delta }}_{jj^{\prime }}}^{prop}\right)}{\sqrt{\frac{1}{n_{j}n_{j^{\prime }}}+\frac{N_{off}}{n_{j}n_{j^{\prime }}\left(N_{diag}\right)}}}\right)}^{2} \end{array}$$

where $\bar{\delta_{W}}$ is the overall mean values of all the dissimilarity values in the partitioning groups $\sum_{\alpha }\sum_{\beta }\delta_{\alpha \beta }\epsilon_{\alpha \beta }/\sum_{\alpha }\sum_{\beta }\epsilon_{\alpha \beta }$ and $\epsilon_{\alpha \beta }$ is an indicator function and is equal with 1 if both α and β observations are in the same group and 0 otherwise.

The $F_{nm}$ formulated in Equation 1 is encapsulating the ratio of divergence of the mean of *between* to *within* groups. Under null hypothesis of no difference between the mean values of the partitioning groups, the sum of normalized means of dissimilarity values *between* each partition would be the same as their *within* counterparts (Data S2). To the degree of deflection from this ground truth, the observed $F_{o}$ would be inflated and so the $\mathrm{Pr}\left(F_{g^{2}-g,g-1}>F_{o}\right)$ quantity would be the assigned p− value to indicate whether this deflection is of any significance with a designated α− level threshold. Table 1 summarizes the essentials of this model with respect to the metric ones.

**Table 1:** Summary of different ANOVA models

Method	ANOVA	Nonparametric	Nonmetric
Specification	Responses	Distances	Dissimilarities
Input	**y**	$D_{N\times N}$	$\Delta_{N\times N}$
Test statistic	$\frac{{SS}_{B}/g-1}{{SS}_{W}/N-g}$	$\frac{{SS}_{B_{d}}/g-1}{{SS}_{W_{d}}/N-g}$	$\frac{{SNM}_{B_{\delta }}/g\left(g-1\right)}{{SNM}_{w_{\delta }}/g-1}$
p value	$P\left(F_{g-1,N-g}>F_{0}\right)$	$\frac{\sum_{\pi }1_{\left[pF_{np}^{\pi }\geq pF_{0}\right]}\left(\pi \right)}{\sum_{\pi }1}$	$P\left(F_{g^{2}-g,g-1}>F_{0}\right)$

The high-level similarity of nmA to cA is its closed form distribution of the Test statistic leading to the respective p value, while the matrix structure of the Input is common between npA and nmA, albeit former based on the Distances (*D*), and Dissimilarities (Δ) the latter (Data S1–S3).

### General properties of nmA

Deriving the test statistic formulated in Equation 1, we are focused on the performance of nmA in different scenarios. This section is providing the basic properties of this statistic under diverse hypothetical cases mediated with multiple input structure and underlying parameters.

#### nmA is sensitive to groups differences

**Figure 1:**
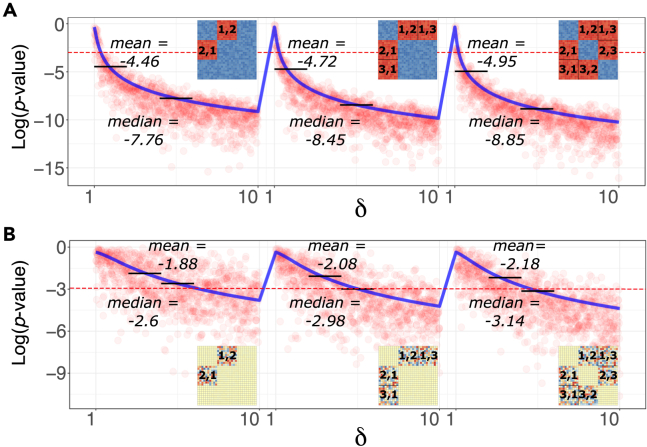
The consistency of the $F_{nm}$ under different scenarios (A) *The effect of progressively changing the mean of the between group matrices distribution on the* p *values.* We use the previous group dissimilarity matrix as our starting point and continuously change one of the uniform distribution parameters via adjusting *ψ* factor (as an incremental adjustment of the *a* and *b* in U(a,b)). With every step *i* from [1,10], we change the *between* group matrix distributions to be $U\left(0+\psi_{i},1+\psi_{i}\right)$ and obtain the corresponding p value. At the same time, we track the effect of the proportion of *between* group data being affected via *ψ* parameter. The three scenarios are visualized (from left to right): when only group 1 and group 2 pairwise *between* group matrices are adjusted; when group 1 and group 2 but also group 1 and group 3 dissimilarity matrices are simultaneously adjusted; when all the 6 possible *between* group matrices are simultaneously adjusted. The red dots represent each p value with corresponding ψ and the mean values of them are connected with the solid blue lines. The red dashed line corresponds to the p value equal to 0.05. (B) *The effect of progressively changing the standard deviation of the between group matrices distribution on the* p *values.* While in previous case the difference in *between* group distributions was triggered via changing the mean of uniform distribution, here we fix the mean but change standard deviation via adjusting the *between* group distribution according to $U\left(0-\psi_{i},1+\psi_{i}\right)$. The adjustment is performed for the same three scenarios as previously. As indicated with the lower values (of the mean and medians) and the higher gradient of descent (solid blue lines), nmA is more sensitive with the dislocation of the mean as the first central moment of the values than the dispersal standard deviation parameter.

As one of the main functionality of a test statistic, we first focused on the sensitivity of nmA in response to incremental changes between mean discrepancy of *between* dissimilarities in relation to *within* (Figure 1A). This is a clear deviation from the nmA null hypothesis and so the incremental shifting has led to the expected inflation of the $F_{o}$ statistics in nmA model (Figure 1A). We were also interested in assessing the degree of robustness of nmA in face of increasing the dispersion of the input. Despite sensitivity of nmA to the location parameter, the scale changes were not leading to the same significant effect as increasing the standard deviation of *between* to *within* groups does not lead to the marked changes in the nmA judgment, denoting the robustness of the method with respect to scale dimension of the inputs (Figure 1B).

**Figure 2:**
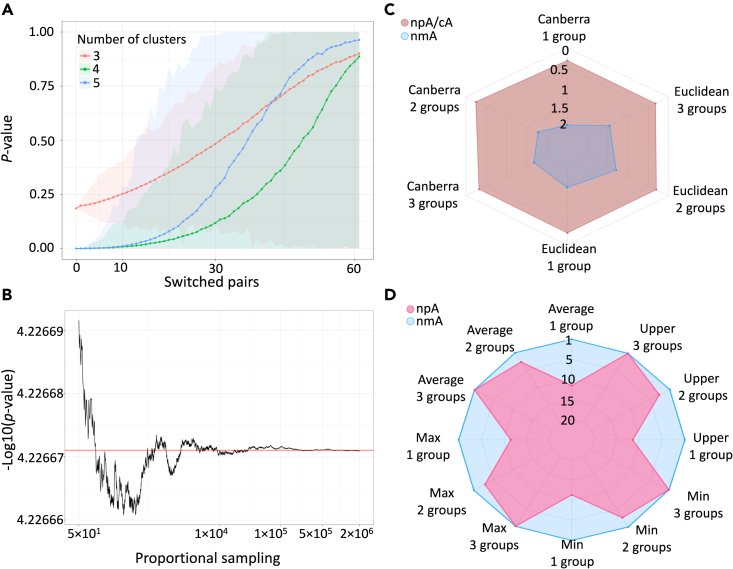
nmA properties and applications (A) *Distribution of* p *values to define optimal clustering.* The original dissimilarity matrix clustering of size 4 has been rearranged to size 3 and 5 to demonstrate that nmA’s p value can serve as an indicator for selecting the optimal clustering of the data. With simulating the dissimilarity matrices of 13, 21, 34, and 55 sizes, we split the matrix into 3 groups via joining the first two clusters, or 5 groups via splitting the last partitioning into 2 groups of sizes 21 and 34. We fix *within* and *between* group dissimilarities distributions to N(0,1) and (5,1), respectively. The choice of Fibonacci sub-sequence for the matrix sizes allows the minimal inclusion of the *between* elements to the *within* when joining or splitting the clusters. Fixing the number of clusters to 3, we obtain clusters of 34, 34, and 55 sizes. This leads to 2∗(13×21) *between* cluster 1 and cluster 2 dissimilarity values coming from N(5,1) distribution mixed with $13^{2}+21^{2}$ dissimilarity values from N(0,1). Similarly, via splitting the cluster of size 55 into 2 clusters of 21 and 34, we set N(0,1) for *between* group dissimilarities. The dotted lines represent the average p values over 1,000 scenarios of switched pairs. The shaded area spans between the minimum and maximum of the p values obtained for each case. (B) *The convergence of* p *values with asymptotic sampling.* The p values converge to their mean value (highlighted in red) after approximately $10^{4}$ proportional samplings performed. (C) *nmA is more conservative with metric inputs.* Four vectors with 100 elements were created using N(10,1) for cA and either Euclidean or Canberra distance was applied to them to obtain 400 × 400 distance matrix for npA. To make nmA applicable, the matrix was transformed via placing the elements of its upper triangular part randomly to the upper and lower triangular parts of a new matrix and taking into account which grouping the elements belong to. The values that have not been imputed in this manner are treated as missing. We further add a small noise from $N\left(0,5\times 10^{-4}\right)$ to all the values of the matrix. After that, the mean of either one, two, or three vectors was progressively increased from 10 to 12. The figure shows the space where a method considers the current data partitioning significant. While cA and npA congruently detect differences at a smaller mean differences, the nmA is exhibiting a more conservative trend. (D) *nmA outperforms cA and npA in nonmetric cases.* The 400 × 400 matrix with *within* and *between* group matrices being from N(30,1) and N(10,1) distribution is used for nmA and it is the coerced symmetric version for npA. The symmetry was achieved via either taking average, minimum, or maximum of the two symmetric counterpart values of the matrix or utilizing the upper triangular part of the created matrix as the input for npA. Similarly to previous case, we start to increase the mean of either one vs. two, two vs. three or three vs. four *between* group matrices while keeping the *within* group matrices the same. This time the result indicates the sharper detection of the differences between the means for the nmA compared with npA.

In addition to its conventional use, nmA can assist in selecting the most similar subclustering of the objects aggregated in a dissimilarity matrix. This application of the method was assessed by tailoring the apparent differentiation between the groups and allowing the model to retrieve that (Figure 2A). Systematically converging the dissimilarities values of the *between* and *within* groups shows an apparent growth of the nmA p value up to the point of saturation mediated by the settled convergence (Figure 2A). Note that here we have used Gaussian distribution since it provides the least negentropy at the constant variance.^29^^,^^30^ The resulting p value trend indicates that no partition can be presented as the most distinct one that was set initially. The implication of this finding allows engineering the nmA for detecting the most optimal clustering between objects in a network with underlying nonmetric mutual dissimilarities.

#### Increased proportional sampling improves accuracy

As the formulation of the $F_{nm}$ is dependent on the proportional sampling entity presented in Equation 1, we were interested in studying the asymptotic proportional sampling behavior of nmA. To assess the convergence of p values with increasing number of the proportional samplings (Data S1), we performed simulations where we collect a sequence of p values which correspond to the same dissimilarity matrix but with a varying number of proportional samplings. Every p value in a sequence is obtained with one more proportional sampling added to the one obtained for a previous p value. For these simulations, we select 3 groups of sizes 10, 20, and 30 and sample dissimilarity matrix from U(0,1) for *within* group matrices and U(2,4) for *between* group matrices. This distribution choice was motivated as the state of uninformativeness in the Bayesian scenarios.^31^ We introduced differences between the groups via increasing mean and standard deviation, and expect p values to reflect it. To check the asymptotic sampling behavior, we applied nmA to the matrix and increase proportional sampling number from 1 to $2\times 10^{6}$. As Figure 2B shows, p values capture the significant difference between the groups and converge to their mean value. This example demonstrates that one can improve the accuracy of a p value via increasing the number of proportional samplings while even a small number of proportional samplings could be conductive of a significant difference.

#### nmA outperforms cA and npA in nonmetric cases

We also considered the comparative performance of cA, npA, and nmA. We note that while npA reduces to cA with the Euclidean inputs,^9^ the relation of the nmA to either cA or npA is not canonical. So any application of the nmA method on the metric data would not be more than a coercion and hence the results would not be comparable nor conclusive. However, we were interested in examining couple of main data modulations that structurally format the date to be a transferable input into different models. The results showed the almost identical sensitivity of the npA and cA models under two different metric measures while the application of nmA on the modulated data led to a more conservative threshold for the rejection of the null hypothesis (Figure 2C). The expected congruency and high sensitivity of the cA and npA models are mainly due to the correct application of these models on the data that are within the space of legit inputs of these models. However, nmA being abused on the metric data, our result underlies a rather more conservative sensitivity with regard to the null hypothesis, such that significant judgment for the rejection of it is always supported with the cA and npA while not necessarily the other way around (Figure 2C).

Tailoring the inputs perfectly for the nmA model and this time coercing the data into metric, we observed an almost inverse phenomenon (Figure 2D). With the nonmetric data, nmA is more sensitive than the npA with respect to rejection of the null hypothesis. We believe the same reason of inadequate compression of the inputs to form a proper input candidate of the npA model is again the main cause of this discrepancy. In effect, this is confirming the merit of each model in the defined sphere of their underlying assumptions with regards to the input data.

#### Asymptotic properties of nmA

**Figure 3:**
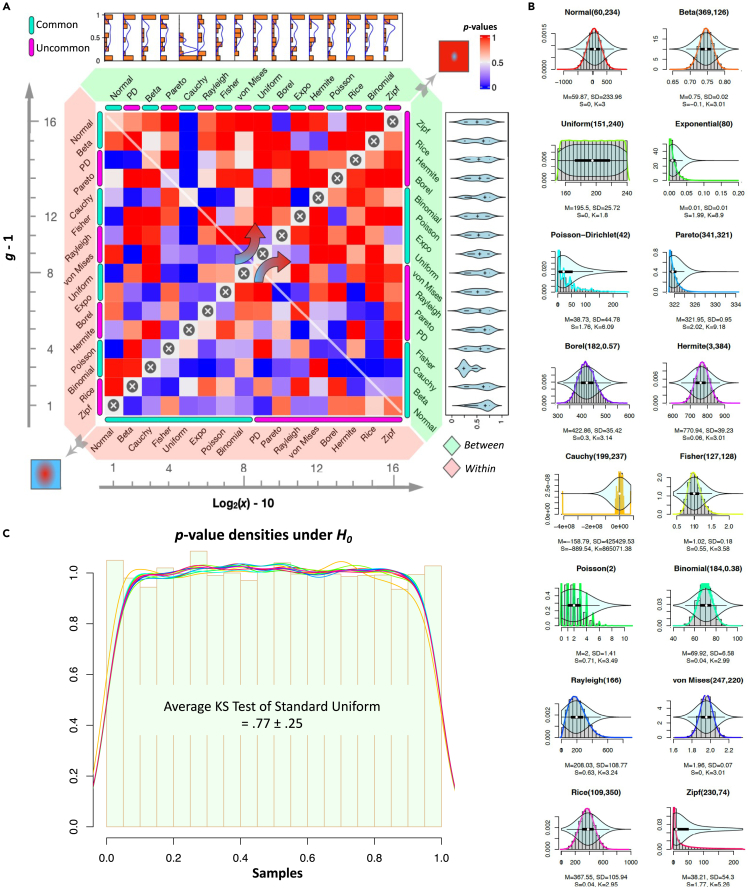
nmA asymptotic behavior (A) *Resolving power of the nmA with increasing number of objects.* Selecting two sets of random variables with closed mathematical form from common and fairly uncommon distributions, we randomly sampled all their respective parameters from 1,400 integer interval (with exception to the *p* parameter of *Binomial(n,p)* and second parameter of *Borel(n,a)* distribution which was normalized by 400 to be <1; and *Uniform(a,b)* with a<b condition). To allow for the maximal information as a result of most numerous comparisons possible, we progressively rearranged the distributions in different orders for each of the four axes of the plot indicated by the narrow flash green and pink colors. On the other hand, we overlaid the light green and light red bands to indicate their respective position on the dissimilarity matrix as either inner sub-matrices (within dissimilarities) or outer sub-matrices (between dissimilarities). The left-to-right axis of the plot is showing the size of the dissimilarity matrix (*x*) and the bottom-to-top axis is indication of number of partitions imposed on the objects (*g*). Note that *x* size of a matrix indicates $\sqrt{x}$ objects and those are randomly grouped in a given *g* number of partitions. For each comparison, the nmA p value is obtained and the marginal violin plots and the densitograms are depicted on the left and the top, respectively. Note that the plot is sectioned with a secondary diagonal line from bottom-left corner to top-right with ⊗ denoting the projection of each value to either right-bottom or top-left axes. The overall trend is leading to insensitivity of the nmA toward the top-right of the plot as opposed to the optimal sensitivity in the lower-off diagonal part of the plot. (B) *Distribution of the variables used to form the asymptotic assay.* The full size sample $\left(10^{8}\right)$ density plots for each variable that was used in the last panel and their respective overlaid violin plot is depicted. Also, the four initial moments as *mean*, *standard deviation*, *skewness*, and *kurtosis* obtained from the sampling procedure is shown with *M*, SD, *S*, and *K*, respectively. (C) *Uniform distribution of the* p *values under null hypothesis.* Obtaining the p values density of the nmA for the 900 sample grouped in three classes of size 10 with placing each of the distributions in former panel as the inner and outer sub-matrices leading to the uniform shape. Colors are preserved from the former panel and the histogram of $10^{3}$ samples from Uniform(0,1) is plotted at the background. The results of mean two-sided Kolmogorov-Smirnov test of uniformity for all the sixteen distributions and its standard deviation are also depicted in the middle of the plot.

One of the important aspects of a test statistic is its asymptotic behavior. To gain more insight about nmA, we performed an omnidirectional survey consisting of multiple distributions, number of objects, and number of partitions. As potentially one can focus on any distribution for data generation, we diversified our survey with choosing sixteen different types of distribution with focus on eight commonly known ones and eight more rare ones such as Poisson-Dirichlet,^32^ von Mises,^33^ Borel and Zipf,^34^ etc. (Figure 3A). The choice of the uncommon distributions was particularly relevant as we are expecting any sort of outcomes as the dissimilarities between objects in the nonmetric space. The other dimension of the survey was increasing the number of the objects (i.e., network size) and the underlying partitions imposed on them (i.e., clusters). Given the collection of distinct distributions (Figure 3B), we were interested in the resolving power of nmA in undifferentiated case of having the outcomes of one distributions to be the *between* dissimilarities and another being the *within*. As all the parameters of the distributions were randomly generated within a confined range (1,400), we observed the wash-out effect of nmA sensitivity by the increased number of objects in comparison to the number of clusters as outlined holistically by the white diagonal line (Figure 3A). This indicates that in presence of umpteen amount of data, either the distinction between the clusters should be more bold to be captured as significant, or the number of clusters in proportion to the network should grow to allow for the same sensitivity. In this setting, this wash-out effect is mediated with increased loss of signal-to-noise ratio as increased number of objects in relatively small number of clusters is leading to diminishing the *between* dissimilarities in relation to the *within* (Figure 3A). Like for example, Cauchy distribution was leading to still significant results in limiting cases of increased network size or even partitions. Surveying its underlying distribution in Figures 3B is confirming the existence of abundant number of erratically different values (huge variance). This is due to the heavy tails of this distribution and so in relation to other “well-behaved” (confined variance) distributions, in case of Cauchy, this is implying the distinction of the mean values derived from CLT formulation on nmA.

The other important property of a valid test statistic that needs to be observed is its p values total indifferential random behavior under null hypothesis. This is indicating that the obtained p values under null hypothesis should follow the uniform distribution^35^ and failure to prove this fact or observing any discrepancy between the p value distribution under null hypothesis versus standard uniform is devoiding the validity of the test statistic derivation and hence its use. To assess nmA performance in this respect, with using the same distribution for the *within* and *between* sub-matrices, we conducted a series of simulations for each of the distributions in the cohort mentioned previously. Obtaining the p values of the nmA shows the standard uniformity dispersion of them under null hypothesis (Figure 3C).

### Applications of nmA

#### Phylogenetic bipartition assessment with BLAST bit scores

**Figure 4:**
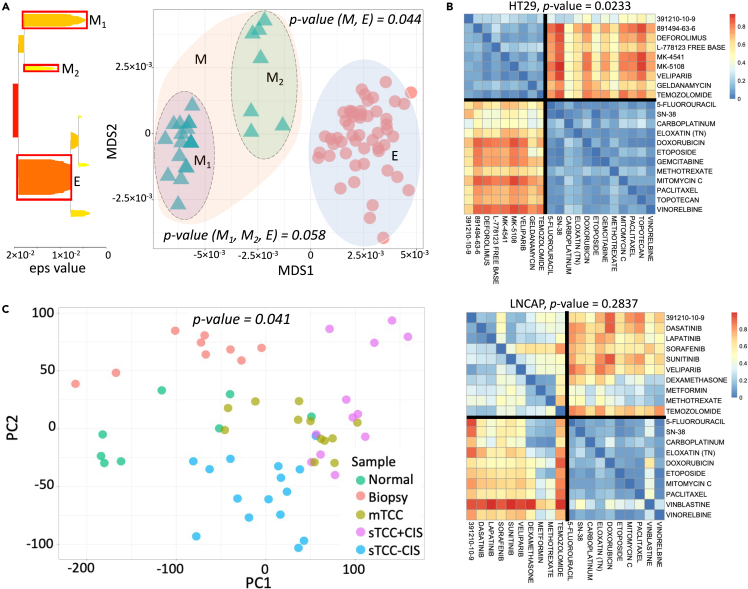
Three diverse biological case studies (A) *MDS and HDBSCAN partitioning of the BLAST data.* The MDS applied on the distance matrix obtained for every species pair as an average of their two nonsymmetric dissimilarities. The HDBSCAN separated the two clusters further via revealing two subgroups in monocots. nmA confirms this grouping with a p value = 0.058, however, suggesting that bipartitioning based on the species is more significant (p value = 0.044). (B) *Heatmap of the most and least* p *values from the drug combination data.* Depiction of the most and least p values for the drug combination clusters suggested by hierarchical clustering with multiscale bootstrap resampling. (C) *PCA-based grouping of the bladder gene expression data.* Some of the sample groups are separated from others while some are rather mixed. nmA provides a significant p value = 0.041 for 5 highlighted partitions as it is sensitive even to one *between* group difference in the data.

For our first example, we consider a set of 78 angiosperms—flowering plants that bear their seeds in fruits.^36^ The data are split into two groups: 26 monocots (one seed leaf plants) and 52 eudicots (two seed leaf plants) (Table S1), leading to a total of ($78^{2}$) 6084 possible pairings. For each pair, we collect basic local alignment search tool (BLAST) output bit scores of the fundamental chloroplast conserved ribosomal protein S8, *rps8* gene.^37^ A bit score is a normalized value of protein sequence similarity provided by BLAST which is a semimetric measure.^38^ Therefore, the cA and npA are not applicable, as neither a base value for a single gene is available (cA requisite) nor the bit scores for angiosperms represent a metric (npA requisite). A higher bit score is a marker of genetic homogeneity between the species, thus inverted bit scores could be used as dissimilarity measurement of their phylogeny. Aggregating these values for all the possible pairs of the species led to the nonmetric matrix of dissimilarities ($\Delta_{78\times 78}$). Considering the partitioning of species into monocots and eudicots imposed on the previously described matrix, we quantify the significance of this bipartition via applying nmA. Our method indicates that the difference between the two species groups is significant with a p value of 0.044, which is in accordance with prior findings in plant physiology as these clusters are dominantly different in their morphological aspects such as leaves, stems, flowers, and fruits.^39^^,^^40^ To assess the existence of any subclusters within the considered groups, we applied HDBSCAN algorithm which constructs hierarchical cluster trees reflecting density estimates together with the stability-based flat cluster extraction.^41^^,^^42^ Based on the algorithm, the most optimal separation is obtained when monocots are further split into two subgroups. Indeed, the separation is noticeable on Figure 4A, where we apply multidimensional scaling (MDS) to the data matrix.^43^ However, the suggested partitioning into three groups is less significant (p value of 0.058) than the one governed by species classes.

Given the dissimilarity matrix, MDS provides the coordinates so that the distances between the points are reflective of corresponding dissimilarities. This result suggests the use of nmA for the phylogenetic bifuricating structure assessment that is often based on nonmetric similarity matrices. The higher-order indices of nucleotide sequence similarities (e.g., bit score, E-value^44^) are obtained from an underlying nonmetric nucleotide substitution matrices. In fact, all the nucleotide substitution models that allow for nonsymetric nucleotide substitution rates (F81, HKY85, TN93, and GTR) will essentially result in nonmetric similarity measure between sequences.^45^ As the metric conditions can be also negated in the case of the codon or amino acids substitution models (such as BLOSUM or PAM), the nmA use can be extended for downstream assessment of partitionings constructed on these model outputs as well.^46^^,^^47^^,^^48^^,^^49^

#### Drug combination sensitivity scoring for cell lines

Combination sensitivity scores (CSS) have been introduced to provide information about drug combination efficacy applied to cancer cells.^50^ After drug sensitivity estimation, it is usually of interest to see whether particular drug groups lead to similar sensitivity patterns. The directional nature of the CSS scores as to which drug combination is a baseline, renders the CSS quasimetric. Consequently, nmA can be applied to any partitioning of drugs to evaluate its significance.

In this example, we apply nmA to assess significance of drug clusters obtained from drug combination experiments. We download relative inhibition (RI) scores (which are sensitivity scores in single drug experiments) and CSS from DrugComb portal.^51^ We filter data to come from O’Neil drug combination study as it contains the most complete dataset.^52^ The data provide RI and CSS for 38 unique drugs and 39 cancer cell lines. For each cell line, we obtain dissimilarity matrices for the 38 drugs, based on the correlations of their CSS_1_ or CSS_2_ values. Afterward, we apply hierarchical clustering with multiscale bootstrap resampling allowing to obtain p values for partitions^53^^,^^54^ on the obtained matrices. We then identify the most significant clusters and apply nmA to retrieve them. To avoid cases when clustering is governed by a small number of drugs (e.g., due to drugs exceptional sensitivity patterns), we apply nmA to dissimilarity matrices that have more than three drugs in each partition. As a result, we identify 9 cell lines out of 14 for which the obtained partitioning is significant at 0.1 level (Table 2 and Figure S1). For most cases, the drugs from two clusters differ by their mechanisms of actions: usually targeted drugs are separated from conventional chemotherapy drugs such as antineoplastic agents, mitotic and topoisomerase inhibitors (Table S2). For example, for HT29, a colorectal adenocarcinoma cell line showing the most significant bipartitioning by nmA, the clustering mostly separates chemotherapy drugs from kinase inhibitors (Figure 4B). It has been reported that approximately 42 tyrosine kinase inhibitors (TKIs) have shown efficacy in colorectal cancers during preclinical studies with regorafenib being the first TKI approved by the Food and Drug Administration.^55^ In alignment to nmA findings, some studies also described the superior efficacy of regorafenib combined with chemotherapy.^56^ We obtain similar partitioning for LNCAP, a cell line derived from a patient with androgen-sensitive prostate adenocarcinoma. However, it is not strongly supported by nmA as there are some distinctive dissimilarities for drug combinations within the same cluster (Figure 4B).

**Table 2:** nmA model specifications for cell lines classified as significant based on CSS data by hierarchical clustering with multiscale bootstrap resampling

Cell line	N drugs	*F*-score	p value
HT29	9, 12	916.8	0.023
ZR751	10, 8	575.3	0.029
NCIH23	4, 11	321.9	0.039
KPL1	7, 9	211.1	0.049
SKMEL30	9, 12	166.5	0.055
MDAMB436	13, 18	154.6	0.057
A375	13, 21	145.4	0.059
COLO320DM	8, 6	67.2	0.086
DLD1	13, 19	50.6	0.099
EFM192B	6, 13	46.4	0.103
A2780	13, 16	28.5	0.131
RPMI7951	13, 25	26.3	0.137
HCT116	5, 25	8.4	0.238
LNCAP	10, 10	5.7	0.284

#### Kullback-Leibler divergence for gene expression data

For our next example, we consider bladder cancer gene expression data obtained for 57 patient samples that were separated into 5 groups: 12 samples representing superficial transitional cell carcinoma with surrounding carcinoma *in situ* (sTCC+CIS) and 16 samples without surrounding carcinoma (sTCC-CIS), 12 samples within muscle invasive carcinomas (mTCC), 8 samples of normal bladder cells from healthy patients (normal), and 9 histologically normal samples (biopsy) close to the carcinoma *in situ* location.^57^^,^^58^ We aim to apply nmA to check if the difference *between* the sample groups is supported with the gene expression-based dissimilarity matrix. To capture the full distributional level of proximity between the gene expressions, we used the Kullback-Leibler (KL) divergence.^59^ The KL divergence represents a measure of how one distribution differs from another referred to as reference distribution. It is preferred over commonly known point-based methods of differential gene expression such as t test as it is providing a more comprehensive level of information regarding the dispersion of the expressions. We first create a dissimilarity matrix via applying KL divergence scoring on the bladder cancer gene expression data. We apply unsupervised hierarchical clustering to the dissimilarity matrix (Figure S2) and principal component analysis (PCA^60^) to the raw gene expression counts (Figure 4C) to check if KL-based clustering is in accordance with PCA grouping. Being a low-dimensional representation of the patient samples from a gene expression data, PCA reflects the variation between the samples via distances on the Figure 4C. At the same time, we obtained a symmetrized and smoothed version of KL divergence, Jensen-Shannon divergence, and applied npA to compare the consistency in significance between the two methods.

Surprisingly, the comparison revealed differences in many scenarios of significant testing (Figure S3). The both methods agree on nonsignificant partitioning of biopsy and normal samples and their significant distinction from the other three sample groups. The other grouping scenarios were marked as significant by npA only. The initial partitioning of the bladder gene expression data into 5 groups is significant for both methods with nmA p value of 0.041. This underlies the effect of different data processing to accommodate for each method, which in turn results in distinct obtained p values. We note that due to this incongruency in the data input, one should be cautious in relating the obtained p values or applying the parallel threshold for rejection of the null hypothesis across different methods.

## Discussion

We introduced a generic type of nmA method that is based on the dissimilarities between a set of objects. These dissimilarities are not needed to be defined with a metric function nor produced systematically. As long as a numeric value representing the closeness of a set of items subject to different partitions is given, the nmA could be applied for statistical quantification of the significance of the underlying partitioning. In cases where the clear partitioning of the data is not obvious or given *a priori*, the permutative use and search for the lowest p value could lead to the most distinctive possible partitioning of the data such as the use case presented as the drug CSS example. In general, this method could be harnessed for quantification of any sort of clustering of a set of objects into different classes as explored in the first example, where it shows the possibility of the nmA for assessing the meaningfulness of a derived bifurcating structure on a phylogenetic tree.^61^ The other use could be on the network graphs where a specific multipartitism with an underlying dissimilarity matrix is given. As pointed in the last example, the nmA can provide a statistical testing for the patient stratification with reference to their gene expressions proximity. The same method of quantification could be applied for labeling the level of distinctiveness of cells in the UMAP or t-SNE plots based on the single-cell differential RNA expressions measured with nonmetric methods such as KL divergence.^62^^,^^63^ The relative performance of the nmA to existing methods including cA and npA also needs to be properly acknowledged. While the cA is solely constructed on the response values and supported by parametric assumptions such as normality of the residuals,^7^ the npA is formulated on the distances between the responses, free from parametric assumption but still confined to the metric conditions. Similar to npA, nmA is constructed on the dissimilarity measures between responses and devoid of any metric assumptions. Based on the CLT, the closed distribution of the test statistic for nmA is obtained, allowing a parametric derivation of the p value. While proportional sampling could provide a more robust output for the nmA, we note that the comparative analysis across the discussed models is still primarily biased as lack of a totally objective method in transforming the data to fit perfectly to each model (Table 1). While these transformations are creeping the obtained results rendering the comparative task ineffective, the retrieval of the responses themselves (as needed for cA) is impossible even with the availability of the dissimilarities or distances between objects (Figures 2D and Table 1).

### Limitations of the study

We also note the limitation of the nmA in face of growing number of partitions in relation to the number of observations. This is leading to quadratic growth of the *between* samples values with regard to *within*. While the degree of the freedom division in $F_{nm}$ formulation is accommodating for this, the different rate of convergence of the underlying means applied in the CLT is creeping the results toward a noncentrality of the *within* mean square error leading to a more false negative judgment toward rejecting the null hypothesis (Data S1). Similar phenomenon could be observed in the case of unbalanced partition sizes where the results would be primarily affected by the biggest partition. In such cases, a weighted sampling can be a solution; however, in extreme unbalanced scenarios this may not be tractable. Furthermore, the curse of dimensionality aroused in multivariate ANOVA and the unbalanced sampling are also challenging scenarios for cA that need to be properly addressed specifically in each experiment^64^^,^^65^ and likely so, nmA could be both developed and adopted in similar scenarios. Taken together, we provide nmA as an attractive method for a variety of scientific questions which involve the assessment of a given partitioning from nonmetric dissimilarity measurements.

## STAR★Methods

### Key resources table

**Table:** 

REAGENT or RESOURCE	SOURCE	IDENTIFIER
**Deposited data**		

Phylogenetic bipartition data	This paper	https://github.com/AmiryousefiLab/nmANOVA/blob/main/phylogenetic_bipartition_data.xlsx https://doi.org/10.5281/zenodo.10011805
Drug combination sensitivity data	DrugComb portal	https://drugcomb.org
Drug combination sensitivity data (filtered to include O’Neil data only)	This paper	https://github.com/AmiryousefiLab/nmANOVA/blob/main/drug_combination_sensitivity_data.xlsx https://doi.org/10.5281/zenodo.10011805
Bladder cancer data	R package bladderbatch	https://bioconductor.org/packages/bladderbatch/
Other raw and analyzed data	This paper	https://github.com/AmiryousefiLab/nmANOVA https://doi.org/10.5281/zenodo.10011805

**Software and algorithms**		

nmA algorithm	This paper	https://github.com/AmiryousefiLab/nmANOVA/blob/main/nm_ANOVA.R https://doi.org/10.5281/zenodo.10011805
npA algorithm	R package ‘vegan’	https://CRAN.R-project.org/package=vegan
HDBSCAN algorithm	R package ‘dbscan’	https://CRAN.R-project.org/package=dbscan

### Resource availability

#### Lead contact

Further information and questions regarding the model, codes, and the data should be directed to the lead contact, Ali Amiryousefi (ali_amiryousefi@hms.harvard.edu).

#### Materials availability

This study did not generate new unique reagents.

#### Data and code availability

• All the data for simulation and the three real examples are deposited at Zenodo and are publicly available as of the date of publication, DOI of which is provided in the key resources table.
• All original code has been deposited at Zenodo and is publicly available as of the date of publication. DOIs are listed in the key resources table.
• Any additional information required to reanalyze the data reported in this paper is available from the lead contact upon request.

### Method details

The method introduced in this paper is expressed formally in the hypothesis testing with nmA section above as well as other related details pertinent to each subsection. Further reference to the implementation of the other method used are given throughout the text, supplementary material, and listed in the key resources table.

### Quantification and statistical analysis

All the quantification and analysis in the manuscript is performed in R with internal and external functions as listed in key resources table. The thorough explanation of the statistical procedures and related parameters and downstream quantification are marked in relevant legend of each figures and partially in the main text.

### Additional resources

Next to the permanent link to the codes, the readers are encouraged to follow the updates and improvement on the implementation of the nmA from github.com/AmiryousefiLab/nmANOVA.
