# Supplementary material for: Resolving network clusters disparity based on dissimilarity measurements with nonmetric analysis of variance
Source: iScience. 2023 Oct 28;26(11):108354. doi: 10.1016/j.isci.2023.108354 (PMC10663764; doi:10.1016/j.isci.2023.108354)
Supplement: Document S1. Figures S1–S3, Tables S1 and S2, and Data S1–S3 [file mmc1.pdf]

iScience, Volume *26*

Supplemental information

Resolving network clusters disparity based on dissimilarity measurements with nonmetric analysis of variance

Alina Malyutina, Jing Tang, and Ali Amiryousefi

Resolving network clusters disparity based on dissimilarity measurements with nonmetric analysis of variance

Supplementary Data

S1. Formulating the F_{nm}

For any α and β in range of N number of objects, define $\delta_{\alpha\beta}$ as the outcome of the dissimilarity function $\delta(\mathbf{y}_\alpha, \mathbf{y}_\beta)$ where $\mathbf{y}$ is either a scalar or a vector of the interested response. Collecting all the pairwise dissimilarities in a square matrix $\Delta_{N\times N} = \{\delta_{\alpha\beta}\}$ forms the *dissimilarity* matrix between object as the counterpart of *distance* matrix in npA (S3.2). For each $j' = 1, 2, \ldots, g$ and $j \neq j'$, let's denote $\bar{\delta_{jj}}$ and $\bar{\delta_{jj'}}$ as the mean dissimilarity measures of the diagonal and off-diagonal sub-matrices of the Δ and $\bar{\delta_{jj'}}^{prop}$ as the mean of a portion of dissimilarity measures in all diagonal matrices, which comprises the same fraction of diagonal matrices as the number of elements in the current sub-matrix in all off-diagonal sub-matrices. It is important to note that $\bar{\delta_{jj'}}^{prop}$ represents the means of proportional fractions that contain elements which do not overlap for any combination of j and j'. The latter is needed to ensure independence of the sum elements in equation 1. Under the null hypothesis of similarity of all partition means, the F_{nm} as presented below is following the F-distribution with $g^2 - g$ and $g - 1$ degrees of freedom;

$$F_{nm} = \frac{SNM_{B_\delta}/g}{SNM_{W_\delta}} \quad \sim_{H_0} \quad F(x)_{g^2-g,g-1}, \tag{1}$$

where SNM_{B_δ} and SNM_{W_δ} are the *sums of squared normalized means* of dissimilarity values *between* and *within* partitioning groups defined as;

$$\begin{aligned} SNM_{W_\delta} &= \sum_{j=1}^{g} (n_j(\bar{\delta_{jj}} - \bar{\delta_W}))^2 \\ SNM_{B_\delta} &= \sum_{j=1}^{g} \sum_{j' \neq j}^{g} \Big(\frac{(\bar{\delta_{jj'}} - \bar{\delta_{jj'}}^{prop})}{\sqrt{\frac{1}{n_j n_{j'}} + \frac{N_{off}}{n_j n_{j'} N_{diag})}}} \Big)^2 \end{aligned} \tag{2}$$

where $\bar{\delta_W}$ is the overall mean values of all the dissimilarity values in the partitioning groups $\sum_\alpha \sum_\beta \delta_{\alpha\beta}\epsilon_{\alpha\beta}/\sum_\alpha \sum_\beta \epsilon_{\alpha\beta}$, ϵ is an indicator function defined in 12, N_{off} is the number of elements in all off-diagonal sub-matrices and N_{diag} is the number of elements in all diagonal sub-matrices .

S2. Proof of F_{nm} distribution

Based on the central limit theorem the mean of n_j^2 unknown independently distributed $\delta_{\alpha\beta}$ with population mean μ and variance σ^2 is normally distributed hence,

$$\forall j \qquad \bar{\delta_{jj}} \quad \sim \quad N(\mu, \sigma^2/n_j^2), \tag{3}$$

Normalization of these values lead to,

$$\forall j \qquad Z_j = \frac{n_j(\bar{\delta_{jj}} - \mu)}{\sigma} \quad \sim \quad N(0,1), \tag{4}$$

Now under null hypothesis of the sameness of the competing groups mean with each other $H_0 : \forall j, j' \in (1, \ldots, g), \quad \mu_{jj'} = \mu$, and hence $\bar{\delta}_{11} = \bar{\delta}_{12} = \bar{\delta}_{12} = \bar{\delta}_{22}, \ldots, \bar{\delta}_{gg} = \bar{\delta}_W$, the sum of squared values of the above would be distributed as the $\chi_{(g-1)}$ as,

$$\chi^W = \sum_{j=1}^{g} (\frac{n_j(\bar{\delta_{jj}} - \bar{\delta_W})}{\sigma})^2 \quad \sim_{H_0} \quad \chi_{(g-1)}, \tag{5}$$

This is true since the sums of squared g standard normal random variables is Chi-squared distributed.

Following the same logic for dissimilarities between groups we have,

$$\chi^B = \sum_{j=1}^{g} \sum_{j' \neq j}^{g} (\frac{(\bar{\delta_{jj'}} - \bar{\delta_{jj'}}^{prop})}{\sigma\sqrt{\frac{1}{n_j n_{j'}} + \frac{N_{off}}{n_j n_{j'} N_{diag})}}})^2 \quad \sim_{H_0} \quad \chi_{(g^2-g)}, \tag{6}$$

where in that tacitly we have used the equation of the mean values of the dissimilarities *between* groups to be equal with that one as the *within* groups, and for the same reason albeit unlike χ^W, we are not losing an extra degree of freedom in χ^B. Again with noticing that the division of two Chi-squared distributed divided by their corresponding degrees of freedom is F-distributed we have

$$F_{nm} = \frac{\chi^B/(g^2-g)}{\chi^W/(g-1)} \quad \sim_{H_0} \quad F_{(g^2-g,g-1)}, \tag{7}$$

or with rearranging of the values in the above we have,

$$F_{nm} = \frac{\frac{1}{\sigma^2}\sum_{j=1}^{g}\sum_{j'\neq j}^{g}\left(\frac{(\bar{\delta_{jj'}} - \bar{\delta_{jj'}}^{prop})}{\sqrt{\frac{1}{n_j n_{j'}} + \frac{N_{off}}{n_j n_{j'} N_{diag})}}}\right)^2/g(g-1)}{\frac{1}{\sigma^2}\sum_{j=1}^{g}(n_j(\bar{\delta_{jj}} - \bar{\delta_W}))^2/(g-1)}, \tag{8}$$

that with crossing out the $1/\sigma^2$ and $g-1$ the (1) is obtained. Note that the F_{nm} is capturing the amount of noncentrality incurred by degree of deflection of the *between* mean dissimilarities in contrast to the *within*. To the extend of this deflection, F_{nm} would be proportionally inflated and if such observed value F_o is more than certain degree ($\Pr(F_{g^2-g,g-1} > F_o)$), it stands as the existence of evidences for significant differences between the competing groups.

S3. Essential notes on metric ANOVA

Consider N data points, realizations of a random variable $\mathbb{Y}$, and indexed by $\alpha, \beta, \theta \in \{1 \ldots N\}$. We further define a metric function d, $\mathcal{S} = \{(S,d)\}$ as a set of all metric spaces spanned by a metric $d(S,S) : S \times S \mapsto [0,\infty)$ on its domain set S. Note that the functions in $\mathcal{S}$ are not necessarily surjective. The metric function defines distances between data points, $d(y_\alpha, y_\beta)$. An $N \times N$ distance matrix D expresses all pairwise distances between data points. The distances in matrix D follow a metric if and only if for all $\alpha, \beta, \theta \in \{1 \ldots N\}$ the triplets (α, β, θ) fulfill the triangle inequality $d(y_\beta, y_\theta) \leq d(y_\alpha, y_\beta) + d(y_\alpha, y_\theta)$ (Gower and Legendre, 1986). From swapping the arguments two more equality arise that the collectively with the triangle inequality are defined as metric conditions as:

$$\begin{aligned} \text{I. Identity}: \quad & d(y_\alpha, y_\beta) = 0 \Leftrightarrow \alpha = \beta, \\ \text{II. Symmetry}: \quad & d(y_\alpha, y_\beta) = d(y_\beta, y_\alpha), \\ \text{III. Subadditivity}: \quad & d(y_\beta, y_\theta) \leq d(y_\alpha, y_\beta) + d(y_\alpha, y_\theta). \end{aligned} \tag{9}$$

S3.1. Classic ANOVA (cA)

Consider a data set partitioned into g groups, each containing $n_{j|j=1,\ldots,g}$ items such that $N = \sum_j n_j$ constitute the total number of items. Each item is assumed to be the outcome of a p-dimensional random variable, Y. For the kth dimension, let y_{ijk} denote the measured response of ith object in the jth partition, for all $i = 1, \ldots, n_j$, $j = 1, \ldots, g$, and $k = 1, \ldots, p$. The cA tests for the meaningfulness of the partitioning in terms of the proportion of variance explained by using the *decomposition of sums of squares* which partitions the *total* sums of squares SS_T to *between* and *within* groups sums of squares, SS_B and SS_W, respectively (Montgomery, 2012):

$$SS_T = SS_B + SS_W \quad . \tag{10}$$

In cA the distances are Euclidean. For the sake of simplicity and without loss of generality, we only use one-dimensional response variable in the following, that is, $p = 1$. In this setup the elements of Equation (10) are defined as:

$$\begin{aligned} SS_T &= \sum_{j=1}^{g} \sum_{i=1}^{n_j} (y_{ij} - \bar{y}_{..})^2 \\ SS_W &= \sum_{j=1}^{g} \sum_{i=1}^{n_j} (y_{ij} - \bar{y}_{.j})^2 \\ SS_B &= \sum_{j=1}^{g} \sum_{i=1}^{n_j} (\bar{y}_{.j} - \bar{y}_{..})^2 \ , \end{aligned} \tag{11}$$

where $\bar{y}_{..}$ and $\bar{y}_{.j}$ are overall mean of the responses and mean of responses in the jth group, respectively.

S3.2. Nonparametric ANOVA (npA)

npA considers pairwise distance matrices between objects Anderson (2001). The distance matrix is given by a metric function d on the responses such that $d : \mathbf{Y} \times \mathbf{Y} \mapsto [0, \infty)$, where $\mathbf{Y}$ is a realization of a p dimensional random variable, given by $\mathbf{y} = \{\mathbf{y}_{ij}; \mathbf{y}_{ij} = (y_{ij1}, \ldots, y_{ijp})\} \in \mathbf{Y}$ for all i and j. We are interested in distances that besides their domain, form an element of $\mathcal{S} \ni (Y, d)$. All the outcomes of the distance metric function can be collected into a pairwise distance matrix $D_{N\times N} = \{d(y_\alpha, y_\beta)\}$, where each entry is the outcome of the distance function for y_α and y_β. The applicability of these

two methods depends on their different initial assumptions and the available information from the experiment; assuming the same independent variable, cA analyzes response values themselves while the npA analyzes pairwise distances. The latter is more suitable for cases where defining an absolute value for the responses is much more difficult than defining their relative order.

npA results by using the fundamental relationship underlying the topology of a set of points. Specifically, the sum of squared distances between points and their centroid is equal to the sum of squared inter-point distances divided by the number of points. For the set of N elements this means that $SS_T = SS_{T_{dE}}$, where $SS_{T_{dE}}$ is the mean of the sum of squared Euclidean distances. Analogous to cA, it is possible to partition the sum to *between* and *within* sums of squared distances, $SS_{T_{dE}} = SS_{B_{dE}} + SS_{W_{dE}}$. The values of the latter formula are equal to their counterparts in (10). The result generalizes to any general metric function d, resulting in the decomposition formula (10)

$$
\begin{aligned}
SS_{T_d} &= \frac{1}{N}\sum_{\alpha=1}^{N-1}\sum_{\beta=\alpha+1}^{N} d_{\alpha\beta}^2 \\
SS_{W_d} &= \sum_{\alpha=1}^{N-1}\frac{1}{\eta_\alpha}\sum_{\beta=\alpha+1}^{N} d_{\alpha\beta}^2 \epsilon_{\alpha\beta} \\
SS_{B_d} &= SS_{T_d} - SS_{W_d}
\end{aligned}
\tag{12}
$$

where η_α takes the n_j value if the αth observation is in the jth group and $\epsilon_{\alpha\beta}$ is equal with 1 if both α and β observations are in the same group and 0 otherwise. Similarly SS_{B_d} and SS_{W_d} denote the *between* and *within* means of sums of square distances. Note that deriving the SS_{B_d} is not straightforward but one can simply make use of the decomposition rule of sums of squares and calculate it as above.

S3.3. Hypothesis testing

In cA, significance of a given partitioning is tested by comparing the averaged ratio of the variance *between* to *within*, hence the F-statistic:

$$
F = \frac{SS_B/g-1}{SS_W/N-g} \quad \sim_{H_0} \quad F(x)_{g-1,N-g}, \tag{13}
$$

which under null hypothesis follows $F(x)_{g-1,N-g}$ distribution. We refer to the values of test statistic based on the observed outcomes of the experiments

as F_o. This value then will be compared against the null F distribution to obtain the P-value, $P = \Pr(F_{g-1,N-g} > F_o)$. Rejection of the null hypothesis indicates the presence of at least one group with mean value differing from the others. With the similar indication for the p-value, in npA a pseudo permutative F-statistic is defined as

$$\mathrm{p}F_{np} = \frac{SS_{B_d}/g-1}{SS_{W_d}/N-g} \quad \sim_{H_0} \quad \mathrm{p}seudoF, \tag{14}$$

Indexing each values of $\mathrm{p}F_{np}$ obtained from π permutation as $\mathrm{p}F_{np}^{\pi}$ the P-value can be obtained as $P\text{-value} = \frac{\sum_{\pi} 1_{[\mathrm{p}F_{np}^{\pi} \geq \mathrm{p}F_o]}(\pi)}{\sum_{\pi} 1}$ where $1_{[A]}(x)$ is an indicator function (that is 1 if $x \in A$ and 0 otherwise), and $\mathrm{p}F_o$ is the observed pseudo F-statistic.

Table S1: Set of 78 angiosperms partitioned into 52 eudicots and 26 monocots that was used to form a semimetric matrix of dissimilarities based on the inverse values of the bit scores of *rps8* chloroplast gene for all the possible pairwise comparisons

Eudicots	Monocots
Aethionema grandiflorum	*Acidosasa purpurea*
Ageratina adenophora	*Acorus calamus*
Anthriscus cerefolium	*Agrostis stolonifera*
Arabidopsis thaliana	*Anomochloa marantoidea*
Arabis hirsuta	*Bambusa emeiensis*
Arbutus unedo	*Brachypodium distachyon*
Atropa belladonna	*Ferrocalamus rimosivaginus*
Barbarea verna	*Festuca arundinacea*
Betula pendula	*Hordeum vulgare*
Brassica napus	*Indocalamus longiauritus*
Capsella bursapastoris	*Leersia tisserantii*
Carica papaya	*Lemna minor*
Castaneamollissima	*Lolium perenne*
Citrus sinensis	*Oryza meridionalis*
Coffea arabica	*Panicum virgatum*
Crucihimalaya wallichii	*Phalaenopsis aphrodite*
Cucumis melo	*Phyllostachys nigra*
Daucus carota	*Rhynchoryza subulata*
Eleutherococcus senticosus	*Saccharum hybrid*
Eucalyptus grandis	*Sorghum bicolor*
Fagopyrum esculentum	*Spirodela polyrhiza*
Fragaria vesca	*Triticum aestivum*
Glycine maxs	*Typha latifolia*
Gossypium raimondii	*Wolffia australiana*
Guizotia abyssinica	*Wolffiella lingulata*
Helianthus annuus	*Zea mays*
Hevea brasiliensis	
Ipomoea purpurea	
Jacobaea vulgaris	
Lactuca sativa	
Lepidium virginicum	
Lobularia maritima	
Lotus japonicus	
Manihot esculenta	
Medicago truncatula	
Millettia pinnata	
Morus indica	
Nasturtium officinale	
Nelumbo nucifera	
Nicotiana tomentosiformis	
Oenothera elata	
Olea europaea	
Olimarabidopsis pumila	
Panax ginseng	
Parthenium argentatum	
Populus trichocarpa	
Prunus persica	
Pyrus pyrifolia	
Silene noctiflora	
Solanum lycopersicum	
Spinacia oleracea	
Theobroma cacao	

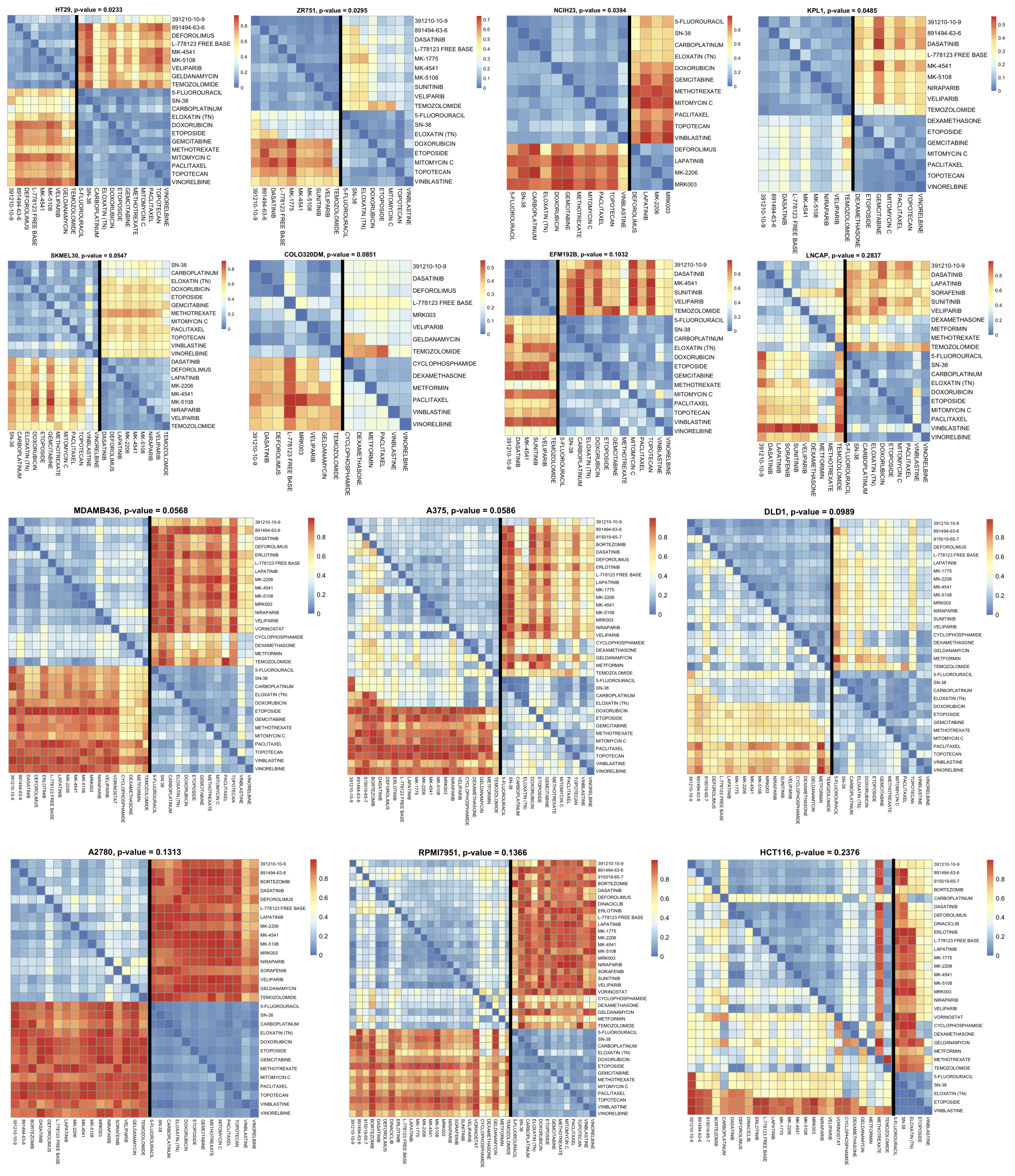

Figure S1: **Bipartitioning of CSS-based dissimilarity matrices for 14 cell lines classified as significant by hierarchical clustering with multiscale bootstrap resampling.** The highlighted bipartitioning of CSS-based specific dissimilarity matrices for cell lines, represented via heatmaps, has been marked as significant by pvclust function. Most of them were classified as significant by nmA (9 cell lines were significant at 0.1 level).

Table S2: Mechanism of action/target and class information for the drugs used in CSS example

Drug	Mechanism/Targets	Class
391210-10-9	MEK inhibitor	Kinase inhibitor
5-FLUOROURACIL	Antineoplastic antimetabolite	Conventional chemotherapy
891494-63-6	CHK1 inhibitor	Kinase inhibitor
915019-65-7	PI3K and mTOR inhibitor	Kinase inhibitor
BORTEZOMIB	Proteasome inhibitor	Protease/proteasome inhibitor
CARBOPLATINUM	Platinum-based antineoplastic agent	Conventional chemotherapy
CYCLOPHOSPHAMIDE	Alkylating agent	Conventional chemotherapy
DASATINIB	Broad kinase inhibitor	Kinase inhibitor
DEFOROLIMUS	mTOR inhibitor	Kinase inhibitor
DEXAMETHASONE	Glucocorticoid, immunomodulatory agent	Immunomodulatory
DINACICLIB	CDK inhibitor	Kinase inhibitor
DOXORUBICIN	Topoisomerase II inhibitor	Conventional chemotherapy
ELOXATIN (TN)	Platinum-based antineoplastic agent	Conventional chemotherapy
ERLOTINIB	EGFR inhibitor	Kinase inhibitor
ETOPOSIDE	Topoisomerase II inhibitor	Conventional chemotherapy
GELDANAMYCIN	HSP90 inhibitor	Conventional chemotherapy
GEMCITABINE	Antimetabolite	Conventional chemotherapy
L-778123 FREE BASE	FPTase and GGPTase-I inhibitor	Conventional chemotherapy
LAPATINIB	HER2, EGFR inhibitor	Kinase inhibitor
METFORMIN	AMPK activato	Metabolic modifier
METHOTREXATE	Antimetabolite	Metabolic modifier
MITOMYCIN C	Antineoplastic anatibiotic	Conventional chemotherapy
MK-1775	WEE1 inhibitor	Kinase inhibitor
MK-2206	AKT inhibitor	Kinase inhibitor
MK-4541	Androgen receptor modulator	Hormone therapy
MK-5108	Aurora A inhibitor	Kinase inhibitor
MRK003	Gamma secretase inhibitor	Gamma secretase inhibitor
NIRAPARIB	PARP inhibitor	Differentiating/ epigenetic modifier
PACLITAXEL	Mitotic inhibitor, taxane microtubule stabilizer	Conventional chemotherapy
SN-38	Topoisomerase I inhibitor	Conventional chemotherapy
SORAFENIB	Broad kinase inhibitor	Kinase inhibitor
SUNITINIB	Broad kinase inhibitor	Kinase inhibitor
TEMOZOLOMIDE	Alkylating agent	Conventional chemotherapy
TOPOTECAN	Topoisomerase I inhibitor	Conventional chemotherapy
VELIPARIB	PARP inhibitor	Differentiating/ epigenetic modifier
VINBLASTINE	Mitotic inhibitor, vinca alkaloid microtubule depolymerizer	Conventional chemotherapy
VINORELBINE	Mitotic inhibitor, vinca alkaloid microtubule depolymerizer	Conventional chemotherapy
VORINOSTAT	HDAC inhibitor	Differentiating/ epigenetic modifierr

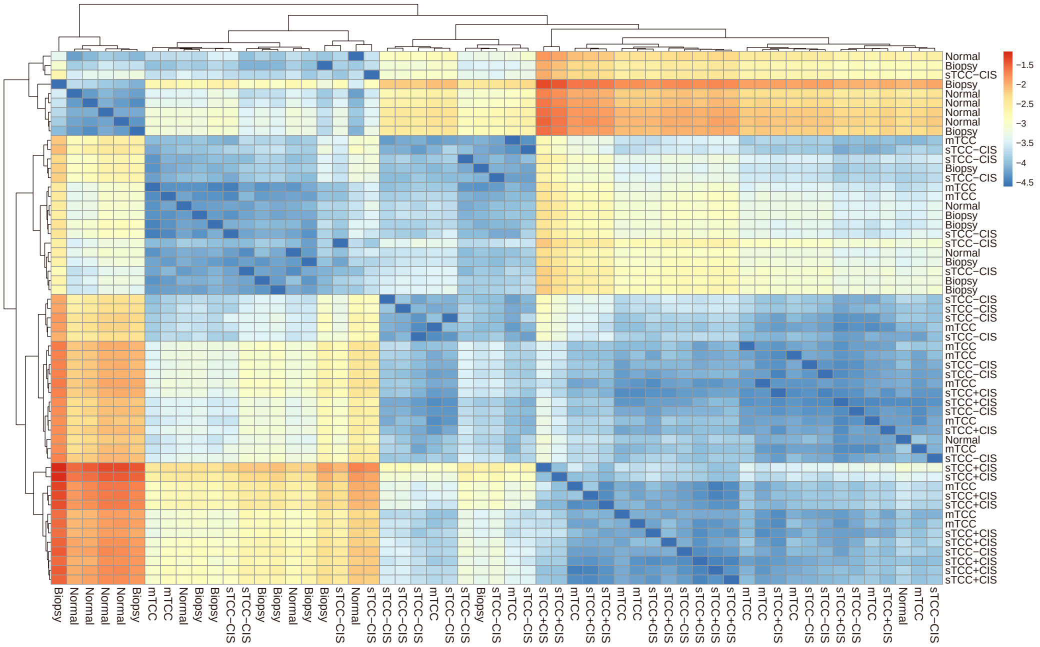

Figure S2: **KL divergence-based dissimilarity matrix for the bladder gene expression data.** The KL-based dissimilarity matrix is visualised via a heatmap of its logarithmic values. We applied unsupervised hierarchical clustering to show relation between the samples. Norml and Biopsy groups show a clear separation from the three carcinoma groups.

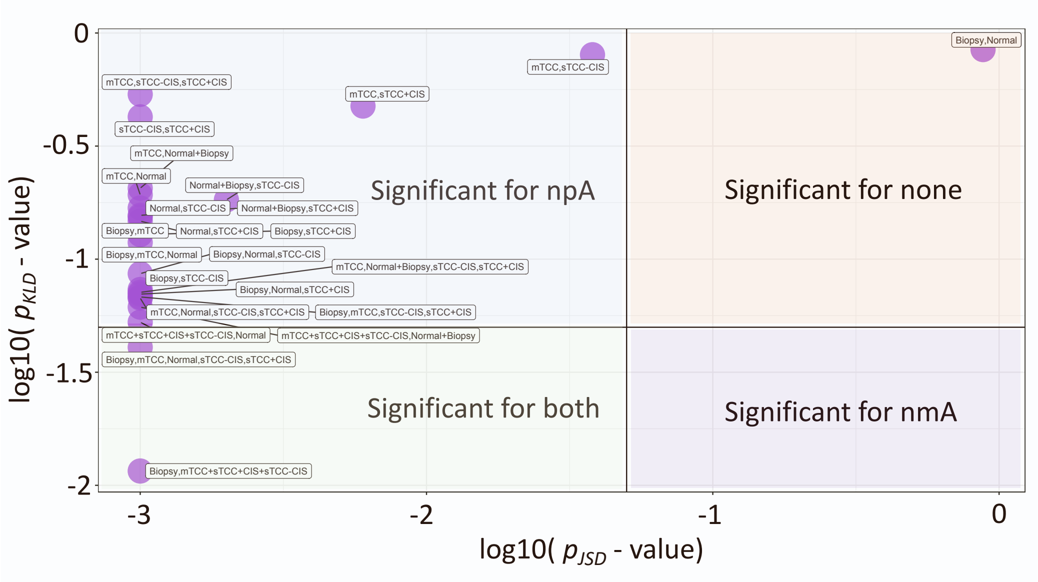

Figure S3: **Comparison of nmA applied to KL divergence (KLD) and npA to JS divergence (JSD) matrices obtained for bladder cancer data.** Both methods, npA and nmA, highlighted non significant partitioning of Biopsy and Normal samples, and their significant distinction from the other three sample groups. The other grouping scenarious were marked as significant by npA only.

References

Anderson, M.J., 2001. A new method for non-parametric multivariate analysis of variance. Austral Ecology 26, 32–46. URL: `http://dx.doi.org/10.1111/j.1442-9993.2001.01070.pp.x`, doi:`10.1111/j.1442-9993.2001.01070.pp.x`.

Gower, J.C., Legendre, P., 1986. Metric and euclidean properties of dissimilarity coefficients. Journal of Classification 3, 5–48. URL: `http://dx.doi.org/10.1007/BF01896809`, doi:`10.1007/bf01896809`.

Montgomery, D.C., 2012. Design and Analysis of Experiments. John Wiley & Sons. URL: `http://www.amazon.com/Design-Analysis-Experiments-Douglas-Montgomery/dp/1118097939%3FSubscriptionId%3D0JYN1NVW651KCA56C102%26tag%3Dtechkie-20%26linkCode%3Dxm2%26camp%3D2025%26creative%3D165953%26creativeASIN%3D1118097939`.
